# Adenovirus Infection in Hematopoietic and Solid Organ Paediatric Transplant Recipients: Treatment, Outcomes, and Use of Cidofovir

**DOI:** 10.3390/microorganisms11071750

**Published:** 2023-07-04

**Authors:** Carlos Grasa, Einés Monteagudo-Vilavedra, Elena Pérez-Arenas, Iker Falces-Romero, Yasmina Mozo del Castillo, Cristina Schüffelmann-Gutiérrez, Teresa del Rosal, Ana Méndez-Echevarría, Fernando Baquero-Artigao, Alejandro Zarauza Santoveña, Pilar Serrano Fernández, Talía Sainz, Cristina Calvo

**Affiliations:** 1 Pediatric Infectious Diseases Department, Instituto de Investigación Sanitaria del Hospital Universitario la Paz (IdiPAZ), Hospital Universitario la Paz, 28046 Madrid, Spain; 2 Centro de Investigación Biomédica en Red en Enfermedades Infecciosas (CIBERINFEC), Instituto de Salud Carlos III—ISCIII, 28029 Madrid, Spain; 3 Pediatric Department, Complejo Hospitalario Universitario de Ferrol, 15405 Ferrol, Spain; 4 Microbiology Department, Instituto de Investigación Sanitaria del Hospital Universitario la Paz (IdiPAZ), Hospital Universitario la Paz, 28041 Madrid, Spain; 5 Pediatric Hematology and Oncology Department, Instituto de Investigación Sanitaria del Hospital Universitario la Paz (IdiPAZ), Hospital Universitario la Paz, 28046 Madrid, Spain; 6 Pediatric Intensive Care Department, Instituto de Investigación Sanitaria del Hospital Universitario la Paz (IdiPAZ), Hospital Universitario la Paz, 28046 Madrid, Spain; 7 Centro de Investigación Biomédica en Red en Enfermedades Raras (CIBERER), Instituto de Salud Carlos III—ISCIII, 28029 Madrid, Spain; 8 Red de Investigación Traslacional en Infectología Pediátrica (RITIP), 28046 Madrid, Spain; 9 Medicine Faculty, Universidad Autónoma de Madrid, Ciudad Universitaria de Cantoblanco, 28049 Madrid, Spain; 10 Pediatric Nephrology Department, Hospital Universitario la Paz, 28046 Madrid, Spain; 11 Pediatric Gastroenterology Department, Hospital Universitario la Paz, 28046 Madrid, Spain

**Keywords:** human adenovirus, hematopoietic stem cell transplant recipient, solid organ transplant recipient, paediatrics, cidofovir

## Abstract

Background: human adenovirus (hAdV) infection constitutes an important cause of morbidity and mortality in transplant recipients, due to their immune status. Among drugs currently available, cidofovir (CDF) is the most prescribed. Methods: Retrospective study of hAdV infection in paediatric transplant recipients from a tertiary paediatric centre, describing characteristics, management, and outcomes, and focused on the role of CDF. Results: 49 episodes of infection by hAdV were detected during a four-year period: 38 episodes in patients that received allogeneic hematopoietic stem cell transplantation (77.6%) and 11 in solid organ transplant recipients (22.4%). Twenty-five patients (52.1%) were symptomatic, presenting mainly fever and/or diarrhoea. CDF was prescribed in 24 patients (49%), with modest results. CDF use was associated with the presence of symptoms resulting in lower lymphocyte count, paediatric intensive care unit admission, and high viral load. Other therapeutic measures included administration of intravenous immunoglobulin, reducing immunosuppression, and T-lymphocyte infusion. Despite treatment, 22.9% of patients did not resolve the infection and there were three deaths related to hAdV infection. All-cause mortality was 16.7% (8 episodes) by 30 days, and 32.7% (16 episodes) by 90 days, of which, 3 episodes (3/16, 18.8%) were attributed to hAdV directly. Conclusions: hAdV infection had high morbidity and mortality in our series. CDF use is controversial, and available therapeutic options are limited. Transplant patients with low lymphocyte count are at higher risk of persistent positive viremias, and short-term survival of these patients was influenced by the resolution of hAdV infection.

## 1. Introduction

Human adenovirus (hAdV) is a double-stranded, linear, nonenveloped DNA virus, classified into 7 different species (A–G). There are 67 different serotypes, but most of the infections in humans are concentrated into 8 serotypes, though this number is higher for serotypes reported in the immunocompromised [1,2,3]. The different serotypes have different tissue tropism, involving a range of areas, from the ocular to gastrointestinal, urinary and respiratory tissues. hAdV is very common in the paediatric population and is involved in a variety of clinical manifestations, which usually resolve spontaneously in immunocompetent hosts after an asymptomatic or flu-like infection. Although rare, severe cases such as myocarditis, encephalitis, and meningitis have been reported, caused by hAdV [4]. However, in paediatric immunocompromised patients, hAdV infection constitutes a major cause of morbidity and mortality: solid organ transplant (SOT) and hematopoietic stem cell transplant (HSCT) recipients are at risk of infection, and hAdV pathogenicity is closely related to the type and stage of host immunity [1,2].

Reactivation and primary hAdV infection are usual events during the immediate post-transplant period and up to six months later: in SOT recipients, this is most commonly described from 1–6 months after transplant [5], and, in HSCT recipients, it is most common during early post-engraftment, from days 30–100 [6]. High-risk patients should be monitored for hAdV infection, since detectable viremia usually precedes the onset of systemic disease [7].

There are important limitations for treating hAdV. Among the drugs currently available, cidofovir (CDF), a cytosine analogue, is the most commonly prescribed [2,7,8], even though its use is controversial, especially in asymptomatic patients. CDF side-effects, such as renal and bone marrow toxicity, are of great concern, and its efficacy remains unclear [9,10,11]. Other options include Brincidofovir, immunoglobulin, and promising strategies such as immunotherapy [9,10]. More conservative approaches involve tapering immunosuppression when possible.

When managing immunocompromised patients, all these strategies should be considered and can be combined, based on risk stratification. However, several critical aspects remain to be determined, such as the optimal viremia cut-off for initiating pre-emptive CDF. Identifying and managing specific groups with less aggressive therapies may also reduce morbidity [12].

This study aims to describe the clinical characteristics of hAdV infection and its therapeutical management and outcome in paediatric transplant recipients. We describe our experience using CDF, in comparison with other series, and analyse current therapies.

## 2. Materials and Methods

### 2.1. Study Design and Population

We retrospectively identified all paediatric transplant recipients who presented a positive hAdV viremia >1000 copies/mL during a four-year period (September 2017 to October 2021) at the reference centre, Hospital Universitario La Paz, in Madrid, Spain.

Weekly systematic viral surveillance was performed for all HSCT recipients during the immediate post-transplant period and up to immune reconstitution. Based on clinical suspicion, SOT and HSCT patients were also tested for hAdV.

Data were obtained from electronic medical records. Extracted information was anonymised and included demographic, clinical, laboratory and microbiological data, lymphocyte count, therapeutic management, and short-term outcome. The study was approved by the Ethics Committee of the hospital, and informed consent was waived, due to the retrospective nature.

### 2.2. Microbiological Testing

hAdV was detected and quantified in whole-blood samples using a quantitative Real-Time PCR assay (RealStar^®^ Adenovirus PCR Kit 1.0, Altona Diagnostics GmbH, Hamburg, Germany). Depending on the situation of the patient, viremia was measured up to twice weekly. In some cases, based on patient’s clinical symptoms, hAdV was also detected in other microbiological samples, such as stool and respiratory samples (nasopharyngeal aspirates or bronchoalveolar lavage).

### 2.3. Definitions

Transplant patients included patients receiving allogenic HSCT or SOT. Patients with a positive hAdV viremia >1000 copies/mL were considered infected (hAdV infection), but only patients with symptoms attributed to hAdV infection were registered as symptomatic patients (hAdV disease), having at least one of the following: fever, respiratory disease, haematuria, and/or involvement of the gastrointestinal tract. Coinfections were determined by reviewing all microbiologic results obtained during the hAdV-positive viremia period. Absence of symptoms or a sustained viremia <1000 copies/mL during a month was considered a resolved infection; a different episode in the same patient was recorded if resolved infection criteria were met. Survival was recorded at 30 and 90 days after the first positive viremia.

### 2.4. Statistical Analysis

Patients’ demographic and clinical characteristics were summarized using counts and percentages or medians and ranges, as appropriate. Two groups of patients were established based on CDF use. Categorical variables were compared using Fisher’s exact test. Continuous variables among groups were compared using the paired *t*-test. To evaluate associations with lymphocyte count, hAdV symptoms, peak viremia, survival, or ICU admission univariant analysis was performed with 95% confidence intervals. All tests were 2-sided, and a *p*-value ≤ 0.05 was considered statistically significant. Statistical analyses were performed using IBM SPSS software (version 21; IBM Corp., Armonk, NY, USA).

## 3. Results

### 3.1. Patient Characteristics and Clinical Manifestations

The study included 49 episodes corresponding to 43 patients, with a median age of 9 years (interquartile range, IQR 4.0–12.0) at the time of first positive viremia. Thirty-two patients (74.4%) had received allogenic HSCT, and 11 patients (25.6%) received a SOT: heart (*n* = 5), small bowel-multi-visceral (*n* = 3), liver (*n* = 1), kidney (*n* = 1), and lung (*n* = 1). Hepatic, renal, or heart insufficiency was present in 14% of patients. Demographic and baseline characteristics of the study population are shown in Table 1; for those patients with more than one episode registered, data shown refers to the first episode of positive viremia.

The estimated incidence of hAdV infection in our centre was 34% (49/144) for all types of HSCT performed.

The median time from transplant to hAdV-positivisation was 85.5 days in the HSCT group (IQR 37.5–217.0) and 180 days in the SOT group (IQR 43.5–1470.5). All patients were receiving at least one immunosuppressive agent, with glucocorticoids (51%), mycophenolate mofetil (26.5%), or calcineurin inhibitors (22.4%) as the most commonly administered. During peak viremia, 25 episodes (51%) presented symptoms due to hAdV disease; symptoms included: febrile syndrome (64%, 16/25), diarrhoea (36%), respiratory distress (32%), abdominal pain (24%), haematuria (16%), rhinorrhoea (12%), rash (8%), and vomiting (4%). Lymphocyte count at the time of hAdV viremia was low, with a median of 395 cells/mL (IQR 120–1245), and there were no statistical differences between lymphocyte counts from asymptomatic vs symptomatic patients during peak hAdV viremia (*p* = 0.8), although lymphocyte count was statistically lower in those who were asymptomatic and received CDF, compared to those who did not receive CDF (*p* = 0.014), Table 2. In 8 episodes, hAdV had been previously reported.

Antiviral prophylaxis was administered to 69.4% of episodes following local guidelines: 46.9% (23/49) acyclovir, 12.2% foscarnet, 6.1% ganciclovir or valganciclovir, and 4.1% other. Coinfection with other virus was detected in 24 episodes (49%), mainly due to cytomegalovirus (CMV) (26.5%, 13 patients) or BK virus (26.5%), but also human herpes virus type 6 (18.4%), Epstein-Barr virus (EBV) (10.2%) herpes simplex type 2 (6.1%), and others (12.2%).

Five episodes showed rejection to transplant (10.2%): 3 HSCT and 2 SOT (1 heart and 1 multi-visceral).

### 3.2. Cidofovir Use, Management, and Outcome

Intravenous CDF was prescribed in 24 episodes (49%). It was administered mainly in the paediatric ward (62.5%), but also at the Paediatric Intensive Care Unit (PICU) (29.1%) and outpatient clinic (8.3%). All episodes presented with symptoms attributed to hAdV (hAdV disease), except for 3 patients, who were asymptomatic but treated, due to risk of progression to disease. CDF was administered at a weekly dose of 5 mg/Kg, with saline pre-hydration and probenecid. Therapy duration was variable, with patients receiving between 1 and 6 doses of CDF (median of 2 doses); the median of duration for those who suppressed viral load was 21 days (IQR 7–36 days), which implied 3 doses (IQR 1–5 doses). Three patients (12.5%) showed renal toxicity attributed to CDF, resulting in 2 episodes to stop the treatment. Out of 24 episodes treated with CDF, 17 (70.8%) improved symptoms that were attributed to hAdV after initiation of treatment, and 16 (66.7%) controlled hAdV infection with virological suppression.

Other therapeutic approaches included recurrent administration of intravenous immunoglobulin (18.3% of episodes, 9/49, with dosing of 0.2–0.8 g/Kg/day, up to 2 g/Kg), tapering immunosuppression (14.3%), and lymphocyte infusions from related donors (12.2%). All these therapeutic options were concomitantly used in patients receiving CDF, except for one.

Of note, despite weekly treatment with 6 doses of CDF, one SOT recipient required complete withdrawal of immunosuppressive agents to control the infection.

As shown in Table 2 and Table 3, CDF use was significantly associated with the presence of hAdV symptoms, low lymphocyte count, ICU admission, and high viral load (peak viremia in patients treated with CDF presented a median of log_10_ 5.3 copies/mL). No association between symptoms’ resolution and cidofovir use could be demonstrated (*p* = 0.17). Clearance of viremia was related to survival outcome, both at 30 and 90 days after infection: 87.8% (36/41) of patients alive at 30 days had suppressed hAdV viremia, compared to 25% (2/8) in the group who died (*p* < 0.001), and 93.9% (31/33) of those who survived at 90 days, compared to 43.8% (7/16) among those who died at 90 days (*p* < 0.001). Both CDF use and persistence of infection were found to be associated with patients’ lymphocyte count at the time of first positive viremia (Table 3).

Despite treatment, 11 out of 24 episodes treated with CDF (45.8%) presented persistent infection and did not recover, showing positive hAdV viremia at the time of their death. This series presented a high proportion of all-cause mortality after infection: 16.7% at 30 days, and 31.3% at 90 days (Table 2). Half of episodes that resulted in death (8/16) were symptomatic by the time of highest viremia detected. In three episodes (18.8%, 3/16), death was attributed to hAdV infection (all before day +30): two episodes from acute liver failure, and one patient with disseminated infection with multiple organ dysfunction syndrome (2 HSCT, 1 cardiac transplant). At the time of the first positive viremia, episodes who died presented a significant lower lymphocyte count (median 120/mcL, IQR 42–630, vs. 680, IQR 235–1500; *p* = 0.042), but not a significantly higher viral load (5220 copies/mL, IQR 1405–43,075, vs 5390, IQR 3025–50,550; *p* = 0.837) compared to those who survived. Among all who died, their last lymphocyte count was notably low (median of 60 cells/mL), with uncontrolled viral load (median log_10_ 8.66 copies/mL).

## 4. Discussion

We report a paediatric series of 43 transplant recipients, both HSCT and SOT recipients, with 49 episodes of hAdV viremia. hAdV infections had high morbidity and mortality in our series, with low lymphocyte count as the main determinant of persistent positive viremia. Both persistent viremia and persistence of hAdV symptoms were associated with short-term survival. CDF was administered in our series in severe episodes, together with other treatments, including more lymphocytopenic and symptomatic patients, with unclear benefits.

Our data support the hypothesis that disease prognosis is poor in immunocompromised patients, as suggested by other authors [2,13]. The incidence of hAdV infections among HSCT in our centre doubles those described in other paediatric series reporting hAdV infection in 14−16% of patients in the early months after HSCT [2]. This fact may be explained by the weekly routine screening that was performed per protocol. According to the literature, the incidence of hAdV viremia in paediatric SOT approaches 8.4–10.1% [14]. However, this study performed surveillance with viral cultures every 2 weeks, which precludes us from comparing with our series those where hAdV was not screened systematically, or where a different technique was used; there is no routine screening for hAdV recommended for SOT recipients [15].

Half of the study participants presented a mild infection or were asymptomatic, and, in those cases, no antiviral treatment was administered. This involvement of hAdV in disease is slightly lower than reported by Fisher et al., who described 56.6% of HSCT recipients that had presented with, or progressed to, hAdV disease [16], and higher than the 37.5% reported by Boge et al., in a cohort of SOT children recipients [17]. As in previous studies, according to our results, hAdV infection correlates with a higher risk of developing hAdV disease and, overall, 90-day mortality.

Due to the retrospective design and the scarce experience and common adverse events related to CDF use, the drug was used in symptomatic and high-risk patients, such as those with a low lymphocyte count, higher peak viremia, or PICU admission, and evaluating the effects is methodologically challenging. Lymphocyte count, concomitant immunosuppressive therapy used, prior hAdV infection status, engraftment, etc. are known factors which may contribute to viral suppression and disease progression, and all were more frequent among patients receiving CDF. Overall, in our series, CDF effect was modest, with 54.2% of episodes treated, resulting in viraemic control, in combination with other therapeutic approaches. Boge et al. reported no development of hAdV disease in 80% (4/5) of SOT recipients with high viremia [17]. On the other hand, there are reports suggesting that pre-emptive therapy with CDF did not decrease the risk of progression to disease or an increase in the viremia in 7 HSCT recipients, compared to 43 that did not receive CDF [16]. According to the literature, and including some older series and case reports, the ability of CDF to control the viremia and to avoid progression ranges from 60 [18] to 98–100% [19,20]. Apart from the challenge of isolating the effect of the different treatments received on hAdV control and disease progression, the risk of bias for reporting successful results should also be considered. To generate data by means of clinical trials is very much needed to create strong evidence to support CDF treatment.

In recent years, brincidofovir has emerged as an interesting therapeutic option against adenovirus: it is a lipid ester prodrug of CDF with reduced renal and bone marrow toxicity and improved pharmacokinetics, having a good oral bioavailability and cell penetration, with a dosing of once–twice per week [21]. A randomised placebo-controlled phase II trial including paediatric and adult recipients of allogeneic HSCT showed that patients that received brincidofovir had a better control of hAdV viremia, with less progression to disease and less mortality than the placebo group, with no renal or bone marrow toxicity [22]. Meena et al. reported a series of 5 children who, after HSCT, received brincidofovir due to uncontrolled hAdV, with good response to therapy and no adverse events attributed to it [23]. However, access to this drug is limited, and, therefore, it cannot be routinely considered for adenoviral infections.

Due to the lack of an optimal antiviral therapy, and, based on the fact that patients unable to resolve the infection are at a higher risk of mortality [12], cellular immune recovery plays a key role in the control of hAdV infection, as suggested by the significantly lower lymphocyte count linked to persistence of infection. Even though CDF could contribute to stabilizing hAdV viremia, virus clearance requires cellular immune reconstitution [18]. This is the rationale underlying the combination of all aforementioned strategies. Specific T cell response correlates with viral clearance [1]. Infusion of intravenous immunoglobulin is routinely recommended in immunocompromised patients, considering the potential of containing high levels of neutralising antibodies against lower adenoviral serotypes [24], commonly co-administered with other therapeutic approaches [25]. Tapering immunosuppression is desirable, but is not always an option. In our series, specific T-lymphocyte infusion from donor was administered in 6 episodes (12.2%). This approach aims to increase T-lymphocytes to control viremia. It is considered, during or after intensive immunosuppressant therapy, to avoid rejection of SOT, or after intensive chemotherapy, while awaiting engraftment in HSCT recipients. Growing interest in cell therapies has been arising in the last years [10,26,27,28]. Interestingly, not only T-lymphocytes, but virus-specific T-lymphocytes (against CMV, EBV, hAdV, etc.), can be administered [27], coming either from the same donor or a third party [29]. Outside the transplant context, this strategy has been used in a premature baby with disseminated hAdV infection [30], and in patients with inborn errors of immunity [31].

CDF was generally well-tolerated in our cohort, with 12.5% of patients presenting with renal toxicity, way below the 31% previously reported [11]. Dosing heterogeneity may account for the differences founded. In fact, other groups reported even lower rates of renal toxicity [16,17,32]. Less common, but also associated to CDF, is bone marrow toxicity, as Sivaprakasam reported in 25% from a cohort of 8 child recipients of HSCT [33], which was not shown in our series. Table 4 summarizes series reporting treatment with CDF in immunocompromised children in the last 5 years, including dosage and administration frequency.

As shown in Table 4, overall morbidity and mortality were high in our series: 31.3% of episodes died (16/49), and 3 episodes were directly attributed to hAdV infection (18.8% of mortality, 3/16; 6.1% of attributed mortality rate, 3/49). These data contrast with those reported by Boge et al., with 100% survival among SOT at 180-day follow-up [17], but are in line with the all-cause mortality rate of 29% described by Fisher et al. among 76 children receiving HSCT (2.4% attributed to hAdV) [16]. The high complexity of our centre may underline the higher rates of mortality shown in our study. As previously reported, resolution of infection was closely related to short-term survival [12,13,15], yet robust data regarding this and other possible risk factors in paediatric transplant recipients are needed. Critical viremia cut-off for mortality needs to be established as well, to determine the optimal threshold for pre-emptive therapy. Any positive viral load [8,34], or a viral load over 10^2–3^ copies/mL [3], have been suggested as possible cut-off points for initiation of therapeutic measures, starting from tapering immunosuppression, but also suggesting antiviral treatment with CDF. As previously reported [13], in our series, where no local guidelines were available, the administration of CDF was not consistent throughout providers, highlighting the ongoing challenge of determining risk factors for disease progression. An effort to create scientific evidence supporting therapeutic options for hAdV infection should be promoted; clinical trials addressing the efficacy of the available management strategies to improve clinical outcomes in high-risk patients are needed.

Apart from this classical indication of CDF in immunocompromised patients suffering from hAdV infection, there are reports where CDF is used in immunocompetent patients, showing controversial usefulness. Alcamo et al. reported the benefits of treating, with CDF, a 3-year-old boy with severe adenoviral sepsis [35], and Christian et al. reported a case where a 2-year-old girl received CDF prior a liver transplant, in a situation of liver failure and high hAdV viremia, which could control viremia and avoid progression to hAdV disease [36]. However, a study in an adult population showed no benefit of treatment with CDF in cases of severe adenoviral pneumoniae [37].

Our study has several limitations, due to the retrospective design, the fact that it was carried out in a single reference centre, the heterogeneity of participants included, and the reduced sample size. Due to the retrospective design, not all the desired variables were available (such as the incidence of bacterial and fungal coinfections) and were not collected. CDF use was not guided by a unified protocol and was often administered with concomitant treatments, impairing our ability to isolate effects. Despite our limitations, we believe that this study adds valuable information regarding hAdV infections and their management, both among children with HSCT and SOT recipients, in which less information is available.

## 5. Conclusions

hAdV infections had high morbidity and mortality in this paediatric series. Patients with low lymphocyte count are at higher risk of persistent positive viremias, and short-term survival was associated with hAdV infection resolution. Cidofovir was administered mainly in severe symptomatic patients, but its benefits could not be established, and identification of patients at higher risk remains a challenge. To validate cut-off values for pre-emptive therapy, and to test the efficacy of management alternatives alone or in combination, is an unmet need.

## Figures and Tables

**Table 1:** Demographic characteristics of the study participants at first episode.

Demographic Characteristics	*n* = 43
Age (median [IQR]) (y)	9 (4.0–12.0)
Sex, female (*n* [%])	22 (51.2)
Clinical characteristics	
HSCT	32 (74.4)
SOT	11 (25.6)
Hepatic, renal, or heart insufficiency	6 (14.0)

Abbreviations: HCST, hematopoietic stem cell transplantation; IQR, interquartile range; SOT, solid organ transplant; y, years.

**Table 2:** Clinical characteristics, therapeutic management, and outcome of all episodes of hAdV infection, and comparison between those treated and not treated with cidofovir, respectively.

Demographics	Total Episodes *n* = 49	Episodes with CDF *n* = 24	Episodes Not Treated with CDF *n* = 25	*p*
Sex, female *	22 (44.9)	8 (33.3)	14 (56)	0.111
Age at the time of positive hAdV viremia (years)	9 (4–11.5)	7.5 (4–11.75)	9 (5–11.5)	0.863
HSCT	38 (77.6)	19 (79.2)	19 (80)	0.791
SOT	11 (22.4)	5 (20.8)	6 (20)	-
Hepatic, renal, or heart insufficiency	7 (14.3)	2 (8.3)	5 (20)	0.417
Time from transplant to positive hAdV viremia (days)	98 (46–295)	56.5 (13–147.25)	175 (81–514)	**0.015**
Asymptomatic patients during peak hAdV viremia	*n* = 24 (49)	*n* = 6 (25)	*n* = 18 (72)	**0.001**
Lymphocyte count (lymphocytes/mL)	595 (183–3063)	185 (107.5–297.5)	920 (257–3382)	**0.014**
hAdV viremia (copies/mL)	3.85 × 10^3^ (1.59 × 10^3^–2.14 × 10^5^)	1.5 × 10^5^ (1.9 × 10^3^–5.3 × 10^6^)	3.6 × 10^3^ (1.47 × 10^3^–8 × 10^3^)	0.242
Symptomatic patients during peak hAdV viremia	*n* = 25 (51)	*n* = 18 (75)	*n*= 7 (28)	**0.001**
Lymphocyte count (lymphocytes/mL)	310 (55–825)	265 (47.5–615)	640 (60–970)	0.177
hAdV viremia (copies/mL)	1.45 × 10^5^ (1.19 × 10^4^–6.86 × 10^5^)	1.9 × 10^5^ (1.3 × 10^4^–4.4 × 10^6^)	3.4 × 10^4^ (8.5 × 10^3^–1.64 × 10^5^)	0.378
**Characteristics in the group treated with CDF**		***n*** **= 24**	**NA**	
Lymphocyte count at the time of 1st CDF administration (lymphocytes/mL)		190 (90–695)	-	
Peak viremia in patients treated with CDF (copies/mL)		1.90 × 10^5^ (4.8 × 10^3^–2.9 × 10^6^)	-	
**Patient location during administration of CDF**		***n*** **= 24**	**NA**	
PICU		7 (29.2)	-	
Paediatric ward		15 (62.5)	-	
Outpatient clinic		2 (8.3)	-	
**Adverse events related to CDF**		3 (12.5)	NA	
Renal toxicity		3/24 (12.5) 3/3 (100)		
Bone marrow toxicity		0		
**Other therapies**	***n*** **= 49**	***n*** **= 24**	***n*** **= 25**	
Immunoglobulin	9 (18.3)	8 (33.3)	1 (4)	**0.011**
Tapering immunosuppression	7 (14.3)	6 (25)	0	**0.01**
T-lymphocyte infusion (memory cells from donor)	6 (12.2)	6 (25)	0	**0.01**
**Resolution of infection and outcome**	***n*** **= 49**	***n*** **= 24**	***n*** **= 25**	
Episode duration (days)	17.0 (7.5–37.5)	29 (8.5–48)	11 (6.5–16.5)	**0.005**
Resolved infection	38 (77.6)	16 (66.7)	22 (88)	0.095
Admission to PICU	7 (14.3)	7 (29.2)	0	**0.004**
**Mortality**	***n*** **= 49**	***n*** **= 24**	***n*** **= 25**	
All-cause mortality after 30 days of infection	8 (16.3)	5 (20.8)	3 (12)	0.464
All-cause mortality after 90 days of infection	16 (32.7)	10 (41.7)	6 (24)	0.187
Death due to hAdV infection	3/16 (18.8) 3/49 (6.1%)	3/10 (33.3) 3/24 (12.5)	0 0	0.25 0.022

* All values expressed as *n* (%) or median [IQR], except otherwise stated. Abbreviations: CDF, cidofovir; hAdV, human adenovirus; IQR, interquartile range; NA, not applicable; PICU, paediatric intensive care unit.

**Table 3:** Univariate analysis of factors associated with CDF use, short-term outcomes, and lymphocyte count.

Cidofovir Use	OR	95% CI	*p* Value
Presence of hAdV symptoms PICU admission Peak viremia > log_10_ 5 hAdV copies/mL	4.57	1.34–16.61	0.020
	1.41	1.09–1.82	0.009
	5.00	1.44–17.27	0.019
**Outcome after resolution of infection**	**OR**	**95% CI**	***p*** **value**
Survival at 30 days	21.00	3.28–134.15	0.001
Survival at 90 days	23.25	3.98–135.68	<0.001
**Lymphocyte count at time of first hAdV viremia**	**Median (lymphocytes/mL)**	**IQR**	***p*** **value**
Cidofovir treatment	622	1470	0.036
No cidofovir treatment	2852	2116	
Resolution of infection	1516	2080	0.002
Persistence of infection	300	505	

Abbreviations: CI, confidence interval; hAdV, human adenovirus; OR, odds ratio; PICU, paediatric intensive care unit; SD, standard deviation.

**Table 4:** Summary of observational cohorts reporting use of CDF in immunocompromised children against hAdV.

Author, Year	Number and Type of Patients	Dosing	Adverse Events	Potential Benefit	Mortality	Comments
Neant et al., 2018 [32]	13, HSCT	5 mg/Kg/weekly (2 doses), then every 2 weeks	Renal toxicity: 1 (7.7%)	Yes (controlling viremia)	1 (7.7%)	12 hAdV, 4 CMV
Fisher et al., 2019 [16]	7, HSCT	5 mg/Kg/week (*n* = 2) 2 mg/Kg/week (*n* = 1) 1 mg/Kg/3 times per week (*n* = 2) 0.25 mg/Kg/2 weeks (*n* = 2)	No data	No	0	Pre-emptive therapy
Boge et al., 2019 [16]	5, SOT	5.5–6 mg/Kg/week	No data	No progression in 4 out of 5 (80%)	0	Pre-emptive therapy
Grasa et al., 2023	24: 5, SOT; 19, HSCT	5 mg/Kg/week	Renal toxicity: 3 (12.5%)	Possible in 54.2%	10 (41.6%) 3 due to hAdV (12.5%)	Concomitant therapies used

Abbreviations: CDF, cidofovir; CMV, cytomegalovirus; hAdV, human adenovirus; HSCT, hematopoietic stem cell transplant; SOT, solid organ transplant.

## Data Availability

Data could be available under reasonable request.
